# Spontaneous Detachment of Colloids from Primary Energy Minima by Brownian Diffusion

**DOI:** 10.1371/journal.pone.0147368

**Published:** 2016-01-19

**Authors:** Zhan Wang, Yan Jin, Chongyang Shen, Tiantian Li, Yuanfang Huang, Baoguo Li

**Affiliations:** 1 Department of Soil and Water Sciences, China Agricultural University, Beijing, 100193, China; 2 College of Land and Environment, Shenyang Agricultural University, Shenyang, Liaoning, 110866, China; 3 Department of Plant and Soil Sciences, University of Delaware, Newark, Delaware, 19716, United States of America; University of Akron, UNITED STATES

## Abstract

The Derjaguin-Landau-Verwey-Overbeek (DLVO) interaction energy profile has been frequently used to interpret the mechanisms controlling colloid attachment/detachment and aggregation/disaggregation behavior. This study highlighted a type of energy profile that is characterized by a shallow primary energy well (i.e., comparable to the average kinetic energy of a colloid) at a small separation distance and a monotonic decrease of interaction energy with separation distance beyond the primary energy well. This energy profile is present due to variations of height, curvature, and density of discrete physical heterogeneities on collector surfaces. The energy profile indicates that colloids can be spontaneously detached from the shallow primary energy well by Brownian diffusion. The spontaneous detachment from primary minima was unambiguously confirmed by conducting laboratory column transport experiments involving flow interruptions for two model colloids (polystyrene latex microspheres) and engineered nanoparticles (fullerene C_60_ aggregates). Whereas the spontaneous detachment has been frequently attributed to attachment in secondary minima in the literature, our study indicates that the detached colloids could be initially attached at primary minima. Our study further suggests that the spontaneous disaggregation from primary minima is more significant than spontaneous detachment because the primary minimum depth between colloid themselves is lower than that between a colloid and a collector surface.

## 1. Introduction

Investigating attachment and detachment of colloids in porous media is of practical interest for various environmental and engineering applications [1–5]. The Derjaguin-Landau-Verwey-Overbeek (DLVO) theory illustrates that the interaction energies controlling the attachment/detachment of a colloid on/from a collector surface include van der Waals attraction, double layer interaction energy and short-range repulsion [6–8]. Summing the aforementioned interaction energies over the separation distance between the colloid and collector results in the so-called DLVO interaction energy profile, which has been frequently employed to predict the attachment/detachment of colloids on/from collector surfaces [9].

**Fig 1:**
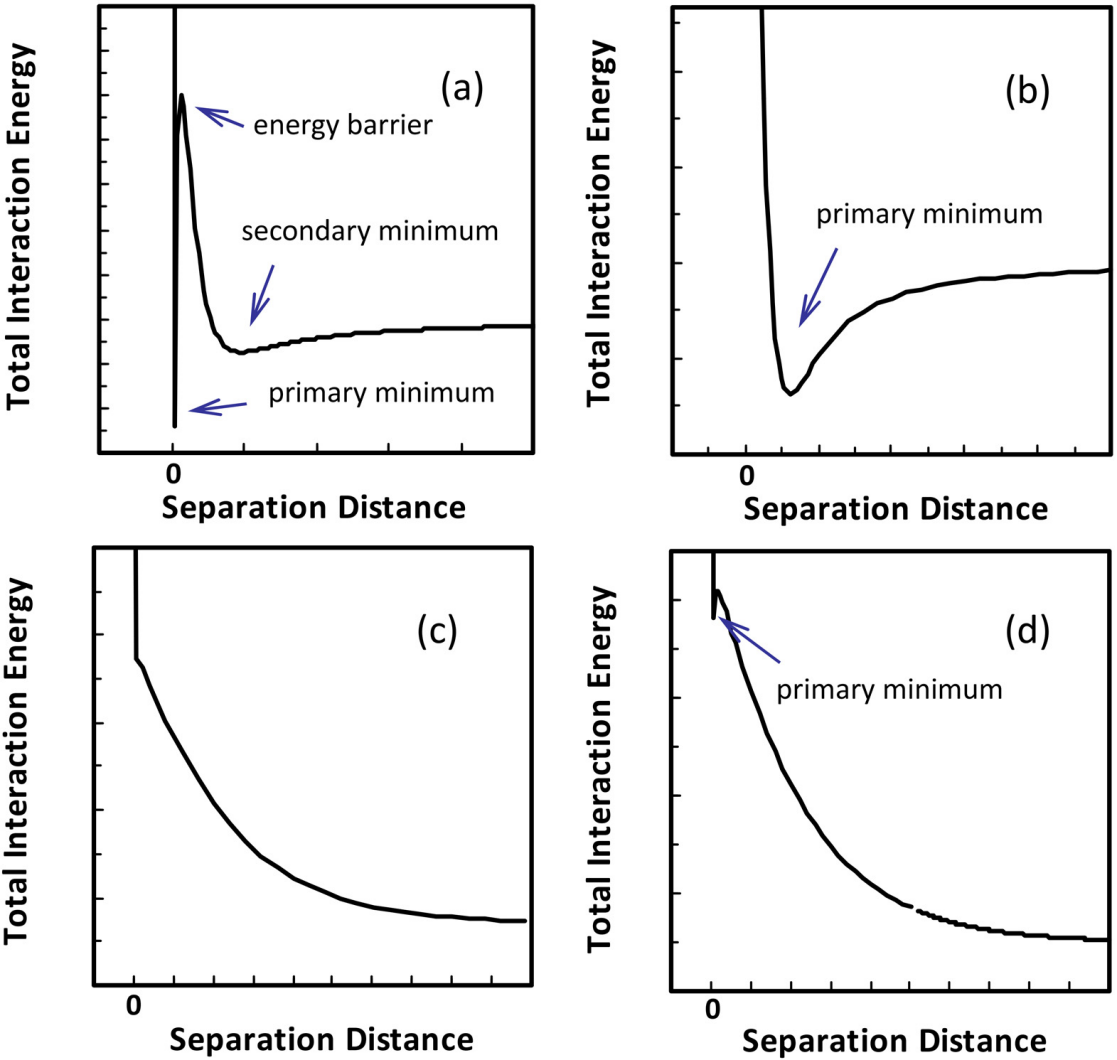
Schematic representations of typic DLVO interaction energy profiles. The DLVO interaction energy profiles in (a), (b), (c), and (d) were denoted as type I, II, III, and IV respectively.

Two typical DLVO interaction energy profiles (EPs) have received considerable attention. Specifically, when the double layer interaction is repulsive, the EP is characterized by a deep primary minimum at a small separation distance, a maximum energy barrier, and a shallow secondary minimum at larger distances (see Fig 1a, denoted as type I). Colloids may be attached at the primary minima by overcoming the energy barrier via Brownian diffusion if the maximum energy barrier is comparable to the average kinetic energy of a colloid (1.5 *k*T, *k* is Boltzmann constant and T is absolute temperature). The colloids may be attached at the secondary minimum [10–13] if the interaction energy barrier is significant and the secondary minimum is deep enough to inhibit colloids from being released into the bulk aqueous phase by detachment forces (e.g., Brownian diffusion and hydrodynamic shear). If the double layer interaction is attractive, only a primary minimum exists in the EP (Fig 1b, type II). This EP indicates that colloids will be attached at the primary minimum due to the attraction.

Recently, Shen et al. [14] theoretically showed that the interaction energy can decrease monotonically with increasing separation distance (see Fig 1c, type III) at low ionic strengths when nanoscale asperities are present. This is because when a colloid is located atop a nanoscale asperity, the weak attraction between the colloid and the asperity can be eliminated by strong repulsion from the collector surface underneath the asperity, leading to the absence of primary minimum in the EP. In this case, the colloid experiences repulsive force at all separation distances, which will be detached from the collector surface. By using this type of EP, Shen et al. [14] theoretically explained the experimental observation [15–19] that colloids attached at primary minima can be detached by decreasing ionic strength.

The current study highlights the presence of a different type of EP (Fig 1d, type IV) through systematically investigating influence of height, curvature, and density of discrete physical heterogeneities on interaction energies. This EP is characterized by a shallow primary minimum (i.e., comparable to the average kinetic energy of a colloid) at a small separation distance and a monotonic decrease of interaction energy with separation distance beyond the primary well. The EP indicates that colloids attached at the shallow primary well can spontaneously detach from collector surfaces by Brownian diffusion even under *constant* physiochemical conditions (i.e., without perturbations in solution chemistry or hydrodynamics). The theoretical calculations were confirmed by results from sand column experiments. These conclusions could explain observations in the literature [1,9–12,16,20–22] that tails were present in breakthrough curves when using deionized water to detach colloids despite the absence of secondary minima in deionized water.

### 2. Theory

Physical and chemical heterogeneities are present on all natural grain surfaces at small scales [23]. To estimate the effects of surface heterogeneities on the interaction energies between colloids and collector surfaces, simplifying assumptions are necessary for modeling of them due to their complexities [14,24,25]. In this study, the physically heterogeneous collector surface was represented as a planar surface carrying an asperity. The chemically heterogeneous collector surface was represented by assigning the asperity with surface charges different from that of the planar surface. This elementary surface heterogeneity model has been widely employed in the literature [13,14,23,25–29].

**Fig 2:**
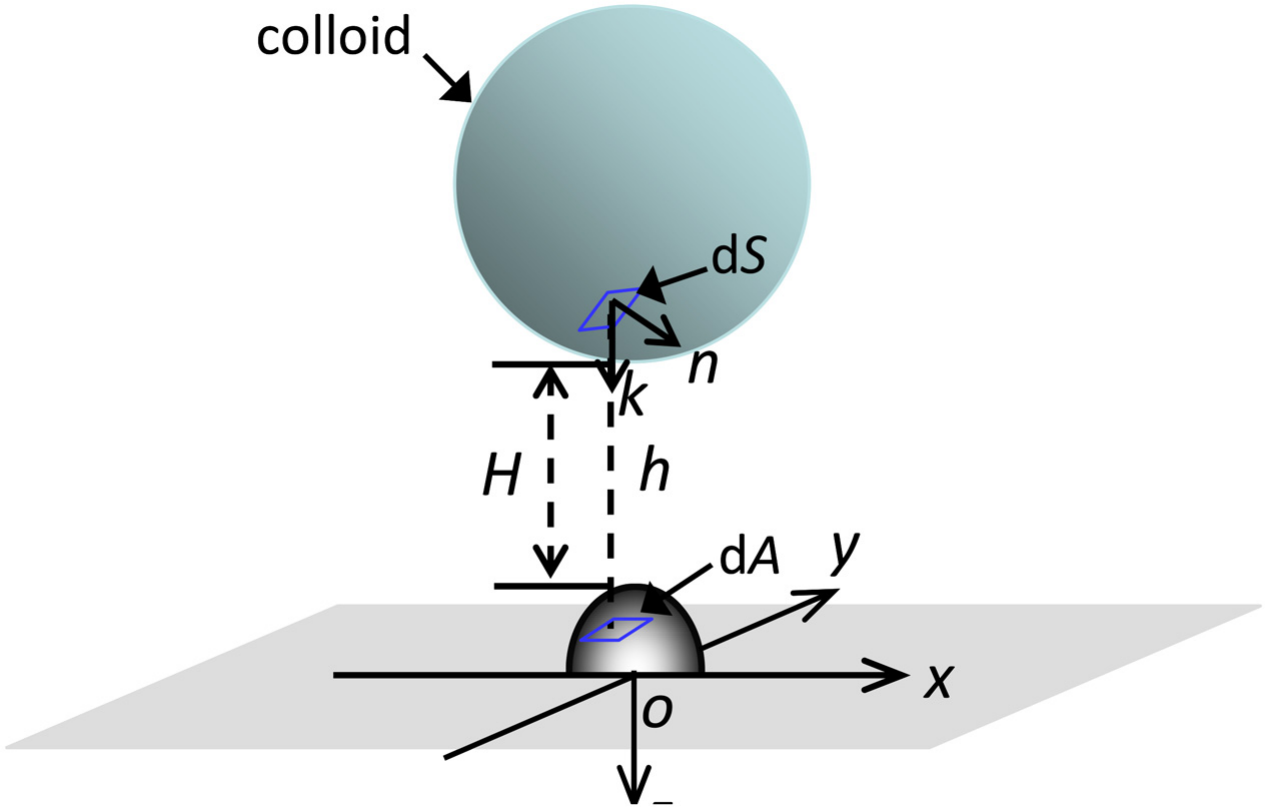
Illustration of a spherical colloid interacting with a planar surface covered with a hemispheroidal asperity. d*S* is a differential area element on the colloid surface, **k** is the unit vector directed towards the positive *z* axis, **n** is the outward unit normal to the colloid surface, d*A* is the projected area of d*S* on the collector surface, *h* is local distance between d*S* and d*A*, *H* is separation distance between the particle and collector surface. Modified from Shen et al. [30].

Fig 2 schematically illustrates a spherical colloid interacting with a planar surface covered with a hemispheroid as a model asperity. The center of the hemispheroid is located directly below the colloid’s center. The asperity was taken as a hemispheroid so that the effects of surface curvature on interaction energies can be taken into account [30]. The grid-surface integration (GSI) technique [31,32] was employed to calculate interaction energies for the interaction configuration in Fig 2. Details about using the GSI technique to calculate the interaction configuration can be found in Shen et al. [30]. Briefly, the Cartesian coordinate system was adopted for the model system. The *xy* plane of the coordinate system is oriented superposing the flat surface. The *z* axis passes through the colloid center and faces away from the colloid. The origin of the coordinate system superposes the center of the hemispheroid. The collector surfaces were discretized into small area elements d*A* by taking the heterogeneities into account. The colloid surface was correspondingly discretized into area elements d*S* related to d*A* by d*A* = (**n∙k**)d*S*, where **n** is the outward unit normal to the colloid surface and **k** is the unit vector directed towards the positive *z* axis. The total interaction energy *U* between the colloid and the collector was obtained by summation of the differential interaction energies over all pairs of element d*S* and element d*A*. The total interaction energy *U* is expressed as

$$U\left(H\right)=\sum_{S}\left(n\cdot k\right)E\left(h\right)\text{d}S$$ (1)

where *H* is separation distance between the colloid and the collector, *E*(*h*) is differential interaction energy between element d*S* and d*A*, and *h* is local separation distance between the element d*S* and the corresponding element d*A*. The value of *h* for each pair of element d*S* and d*A* can be related to *H* in the coordinate system by simple geometric calculations [30,33,34]. The value of *E*(*h*) is calculated by adding van der Waals (VDW) attraction, the constant potential double layer (DL) interaction, and the Born (BR) repulsion [i.e., $E\left(h\right)=E^{\text{VDW}}\left(h\right)+E^{\text{DL}}\left(h\right)+E^{\text{BR}}\left(h\right)$]. The expressions derived by Hamaker [35], Hogg et al. [36], and Oliveira [37] were adopted to calculate *E*^VDW^, *E*^DL^, and *E*^BR^ respectively (see Table A in S1 File). A Matlab program was developed to calculate the total interaction energies for the interaction configuration in Fig 2.

Note that the GSI technique is modified from surface element integration (SEI) technique [38–40]. The SEI technique has circumvented the limitations of Derjaguin’s approximation, which can accurately estimate the interaction energies for nanoparticles or collectors with nanoscale asperities at small separation distances [38]. The GIS technique, by using a different discretization of the surface, can be further applied to calculate interaction energies for surfaces where both physical asperities and discrete charges are present [31,32].

## 3. Materials and Methods

### 3.1. Colloidal particles and porous media

The white carboxyl-modified polystyrene latex colloids (Invitrogen Life Technologies) with a mean diameter of 1156 nm were used. The colloid is hydrophilic with a density of 1.055 g/cm^3^ (reported by the manufacturer). Stock solutions of the 1156 nm colloids were diluted in NaCl electrolyte (Fisher Scientific) to prepare the input solutions for column experiments. The concentrations of the colloids in the input solutions and effluents of the column experiments were determined by UV-vis spectrophotometry (DU Series 640, Beckman Instruments, Inc., Fullerton, CA) at a wavelength of 440 nm using a calibration curve [20].

Quartz sand with sizes ranging from 300 to 355 μm was used as model collector grains for the transport experiments. The sand was sieved from Accusand 40/60 (Unimin Corporation, Le Sueur, MN) with a stainless steel mesh. The procedure from Zhuang et al. [41] was used to extensively remove metal oxides and other impurities from the sand. The treated sand was sonicated in deionized water for 10 minutes and then washed using deionized water until the supernatant was free of colloidal impurities.

A Zetasizer Nano ZS (Malvern Instruments, Southborough, MA) was used to measure electrophoretic mobilities of the colloid and sand in NaCl electrolyte of different concentrations and pHs at 25°C. The finest fraction of sand sieved from Accusand was used for the measurement. The measured electrophoretic mobilities were converted to zeta potentials using the Smoluchowski equation [42]. The measurements were repeated three times for each colloid suspension and the average values were reported in Table 1 and Fig A in S1 File.

**Table 1:** Zeta potentials of 1156 nm colloid, sand, and alumina.

ionic strength (M)	zeta potential (mV)		
	1156 nm colloid	sand	alumina
0.0001	-58.35 ± 4.55	-49.62 ± 4.12	46.8
0.001	-46.73 ± 6.38	-39.27 ± 5.63	36.2
0.01	-46.46 ± 6.51	-39.13 ± 2.45	25.7
0.1	-38.04 ± 4.26	-32.08 ± 3.78	13.4
0.2	-35.21 ± 3.73	-27.49 ± 2.19	8.1

### 3.2. Column transport experiments

The colloid transport experiments were conducted in acrylic columns packed with the cleaned sand. The column was 3.8 cm in diameter and 10 cm long having a similar design as used in Shen et al. [20]. Sand was wet-packed with vibration to minimize air entrapment and to ensure uniformity of packing. The porosities of packed beds were determined to be 0.33 (based on a density of 2.65 g/cm^3^ for the sand).

Column experiments were performed at an approach velocity of 1.2×10^−5^ m/s. For each experiment, background 0.2 M NaCl electrolyte solution (degassed) at pH 10 was first delivered into the column upward for at least 20 pore volumes (PVs) for equilibrating the system. The column was then sequentially delivered with 20 PVs of colloid suspension (10 mg/L) (phase 1) to attach colloids in the 0.2 M NaCl electrolyte, 5 PVs of colloid-free electrolyte solutions (phase 2) to displace unattached colloids in pore water, and 10 PVs of deionized water (phase 3) to detach colloids attached in phase 1. The column experiments have been frequently stopped after phase 3 in previous studies [2,10–12,14,16,17,20–22,30] because it is commonly believed that flushing with deionized water can release all reversibly attached colloids. Our study, however, halted the flow of the column system for 3 days after phase 3 to examine whether spontaneous detachments by Brownian diffusion are present (i.e., phase 4). In phase 5, the column was flushed again with deionized water to elute the colloids detached (if there is any) in phase 4. Note that the ionic strength of the deionized water was changed to about 0.0001 M when its pH was adjusted to 10.

## 4. Results

### 4.1. DLVO interaction energy profiles

The EPs were calculated for the interaction configuration in Fig 2 to predict detachment of the 1156 nm colloids attached at 0.2 M upon reduction of ionic strength in the column experiments. The measured zeta potentials in Table 1 were adopted for the calculations. A value of 1×10^−20^ J was taken as the Hamaker constant for the polystyrene-water-quartz system [12,20,27,28]. We considered the hemispheroidal asperity with various equatorial radii (0–10 μm) and heights (0–1 μm) according to the atomic force microscopy measurement of sand surface’s roughness [43]. We found that four types of detachment could occur due to variation of asperity height and curvature. Specifically, colloids may be irreversibly attached at primary minima therefore do not detach (denoted as type 1), or immediately detached from primary (type 2) or secondary minima (type 3) upon reduction of ionic strength. In addition, colloids may remain attached at primary minima during transient in ionic strength but escape from the primary minima by Brownian diffusion at low ionic strengths (type 4).

**Fig 3:**
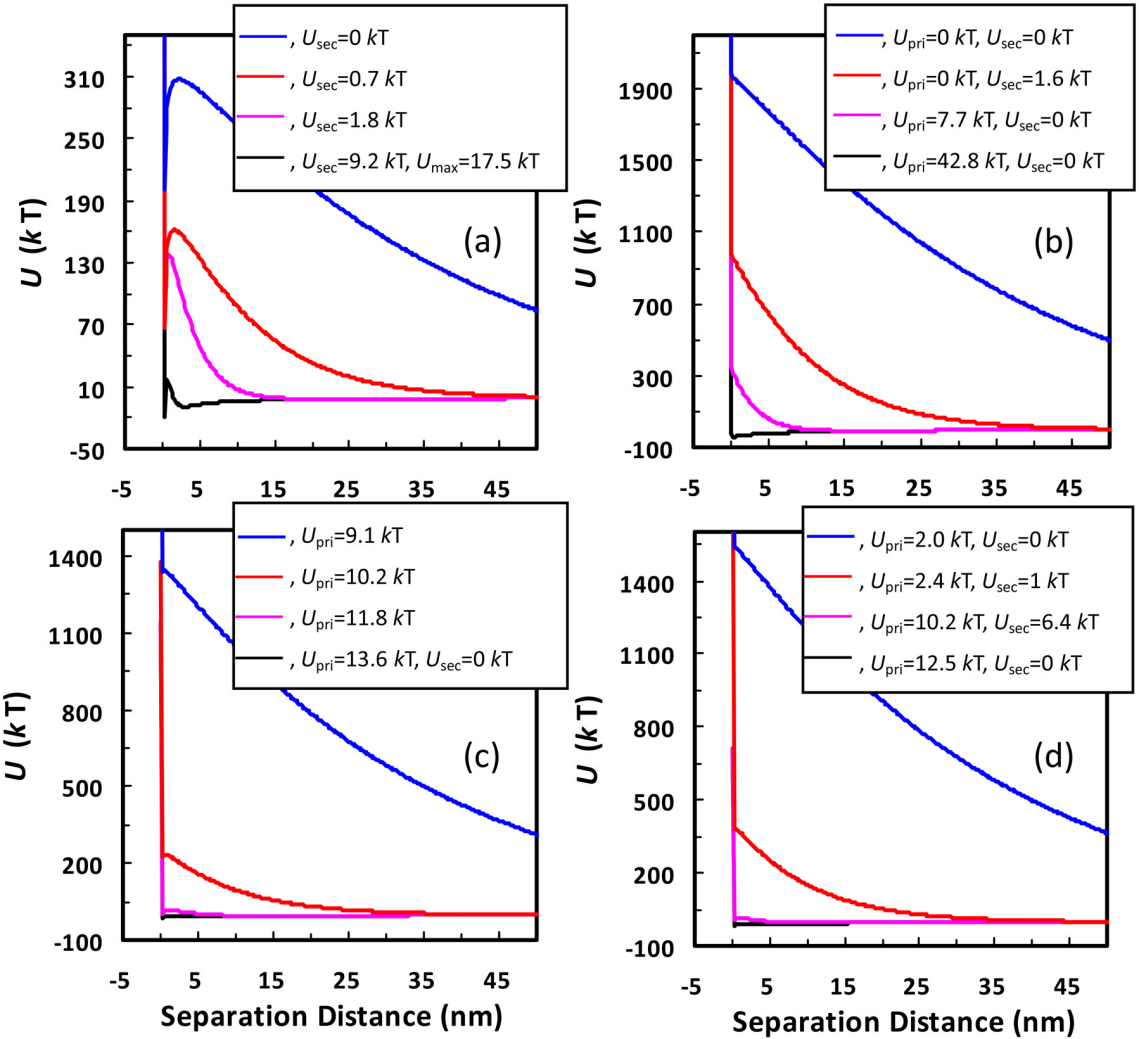
DLVO energy profiles for the 1156 nm colloid interacting with the planar surface carrying a hemisphere with different radii (a, 100 nm; b, 5 nm; c, 20 nm; d, 15 nm) at different ionic strengths (black, 0.2 M; pink, 0.01 M; red, 0.001 M; blue, 0.0001 M). The calculated primary minimum depth (*U*_pri_), maximum energy barrier (*U*_max_), and secondary minimum depth (*U*_sec_) are also shown.

Fig 3 presents four typical sets of EPs for the 1156 nm colloid to illustrate the aforementioned four types of detachment. The equatorial radius of the asperity was taken to be equal to its height (i.e., hemisphere). Each set contains four EPs for a given asperity radius at ionic strengths from 0.2 M to 0.0001 M. In Fig 3a, both energy barrier (*U*_max_, = 17.5 *k*T) and secondary minimum (*U*_sec_, = 9.2 *k*T) are significant in the EP (i.e., type I) at 0.2 M. According to the Boltzmann factor model [$\alpha =\text{exp}(-U_{\text{max}})$, where α is attachment efficiency], only several *k*Ts of energy barrier can essentially inhibits the colloid from being attached at primary minima. Therefore, the 1156 nm colloid is attached at the deep secondary minimum at this ionic strength. The attached colloid will be detached upon reduction of ionic strength because the secondary minimum depth decreases with decreasing ionic strength and eventually disappears from the energy profile at 0.0001 M (i.e., type 3 detachment). In Fig 3b–3d, only primary minima are present on the energy profiles at 0.2 M (i.e., type II), thus, the colloid is attached at the primary minima. The primary minimum depth (or detachment energy barrier from primary minimum, denoted as *U*_pri_) decreases with decreasing ionic strength. Particularly, the primary minimum completely disappears from the EP and the interaction energy decreases monotonically with separation distance (i.e., type III) at 0.0001 M (Fig 3b). Therefore, the attached colloid will be detached from the primary minimum due to repulsion (type 2 detachment). The monotonic decrease of interaction energy with separation distance has been frequently observed in atomic force microscope examinations [44–49]. In contrast, the value of *U*_pri_ (9.1 *k*T) is still significantly greater than the average kinetic energy of a colloid at 0.0001 M (Fig 3c), indicating that the attached colloid is irreversible even at reduced ionic strength (type 1 detachment).

In Fig 3d, although the primary energy well still exists on the EP at 0.0001 M, the energy depth (i.e., 2.0 *k*T) is comparable to the average kinetic energy of a colloid. Beyond the shallow primary well, the interaction energy decreases monotonically with separation distance. This EP (i.e., type IV) illustrates that the colloid may remain attached during transient in ionic strength but will escape from the shallow energy well to bulk solution at 0.0001 M by Brownian diffusion with elapse of time (type 4 detachment). Interestingly, it is easier for the colloid to be detached from the primary minimum of type IV EP than from the secondary minimum of type I EP if the energy depths are similar. This is because the colloid only requires instantaneous forces (e.g., the Brownian motion) to escape from the primary energy well of type IV EP. Once the colloid mobilizes out of the primary well, it will be transported away due to repulsion (as indicated by the monotonic decrease of interaction energy with separation distance). The colloid, however, experiences attractive force from the separation distance of the secondary minimum (to an infinite distance) for type I EP. The attractive force could pull the colloid back into the secondary minimum again.

**Fig 4:**
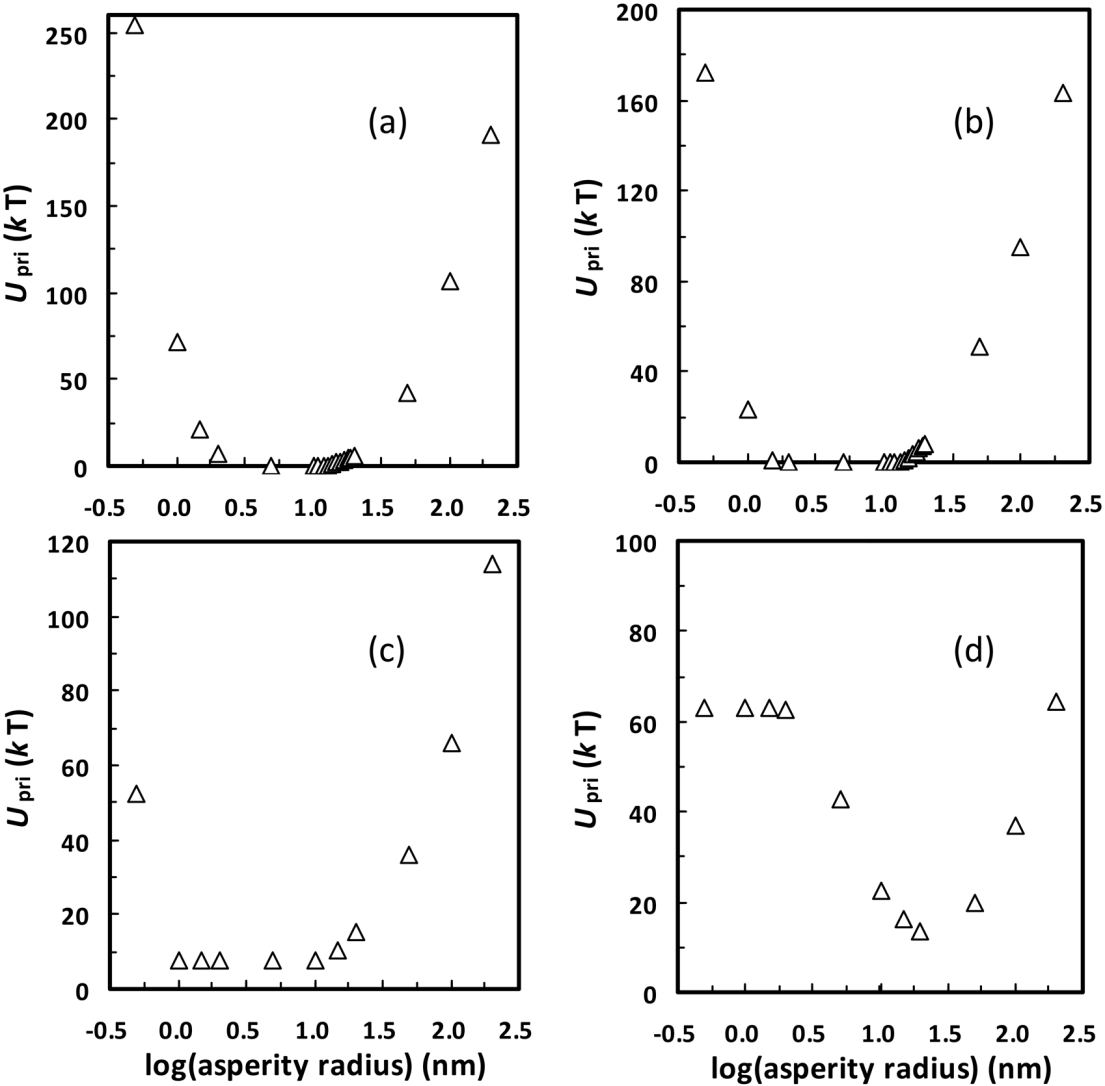
Calculated primary minimum depths *U*_pri_ for the 1156 nm colloid interacting with the planar surface carrying a hemisphere with different radii at different ionic strengths (a, 0.0001 M; b, 0.001 M; c, 0.01 M; d, 0.2 M). Note the change in scale of the *y* axes among the various graphs.

Notably, the spontaneous detachment from primary minima can only occur at low ionic strengths (e.g., ≤ 0.001 M), similar to the spontaneous detachment from secondary minima [26]. Specifically, Fig 4 presents calculated primary minimum depths for the 1156 nm colloid interacting with the planar surface carrying a hemisphere with different radii at different ionic strengths. At a given ionic strength, there is a critical value of asperity radius where the value of *U*_pri_ reaches a minimum, below and above which it increases until reaching the limit (i.e., the value of *U*_pri_ between the colloid and the planar surface). This is because the rough model of Fig 2 becomes the planar surface when the asperity radius is infinitely small or large [43]. At 0.0001 or 0.001 M, there exists a range of asperity radii at which the primary wells are shallow enough (e.g., < 5 *k*T) for the colloid to detach spontaneously. At ≥ 0.01 M, even the smallest primary minimum depths (e.g., 7.8 *k*T at 0.01 M) are significantly greater than the average kinetic energy of a colloid, indicating that spontaneous detachment from primary minima does not occur. However, if the asperity is considered as a hemispheroid, the shallow primary minima are also present at ≥ 0.01 M for large heights (i.e., ≥ 100 nm) and small equatorial radii (see Fig B in S1 File). Although the colloid attached atop these long and sharp asperities can be spontaneously detached from primary minima at ≥ 0.01 M, these asperities rarely exist on natural collector surfaces and the detached colloid is readily re-attached at favorable locations which are widely distributed on collector surfaces at high ionic strengths. Therefore, only at low ionic strengths can the colloid successfully transfer from primary minima to bulk solution by spontaneous detachment.

**Fig 5:**
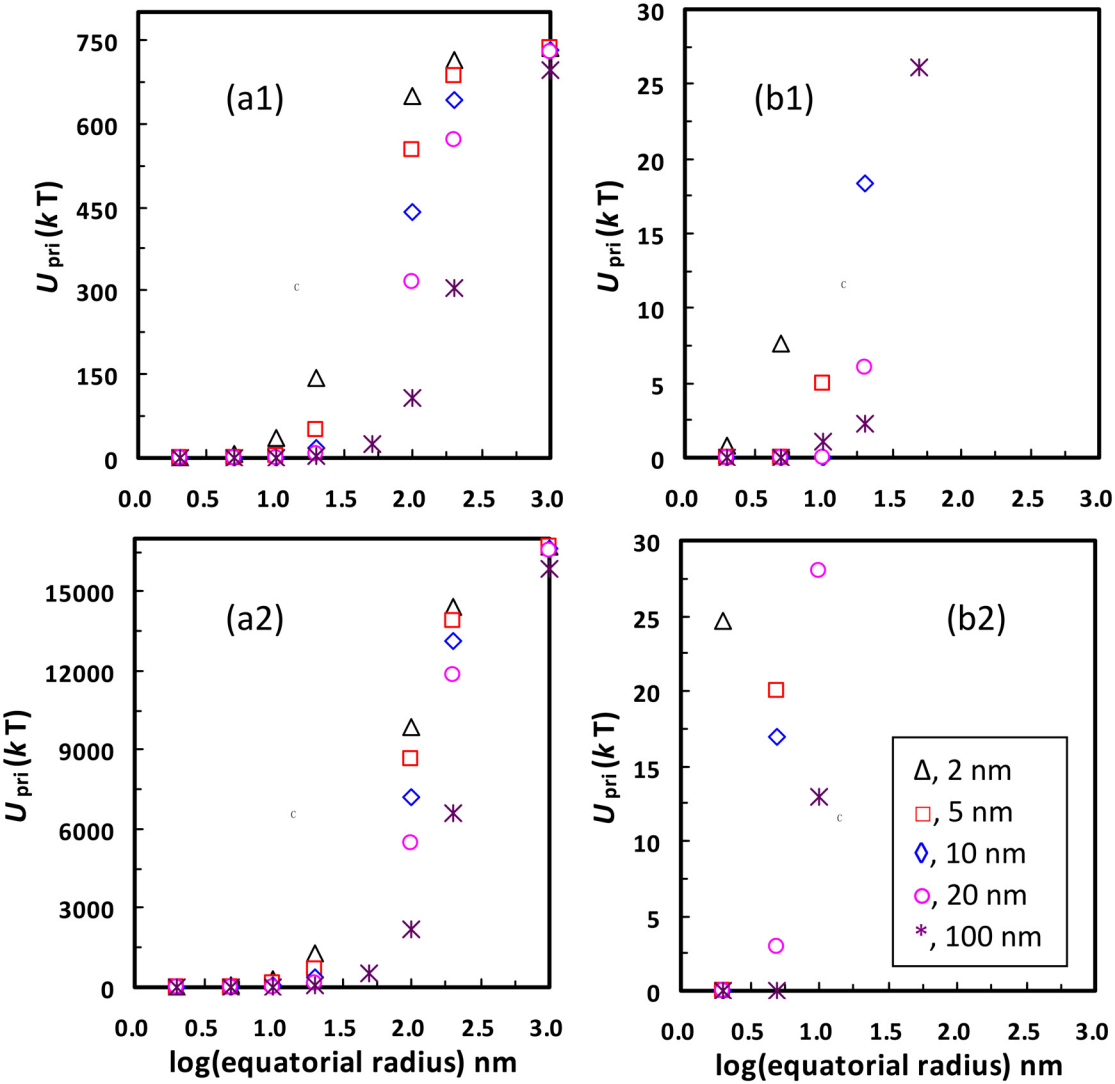
Calculated primary minimum depth *U*_pri_ for the negatively charged 1156 nm colloid interacting with the negatively charged planar surface carrying a (1) negatively or (2) positively charged hemispheroid as a function of equatorial radius for various hemispheroid heights at ionic strength of 0.0001 M. (b) are replotted figures in a different scale of the *y* axis for (a) to highlight the shallow primary energy wells.

While the physical heterogeneity decreases the depth of the primary minimum in EPs, the presence of chemical heterogeneity can increase the primary minimum depth and accordingly inhibit detachment from primary minima. For example, Fig 5 compares calculated values of *U*_pri_ for the negatively charged 1156 nm colloid interacting with the negatively charged planar surface carrying a negatively or positively charged hemispheroid of different equatorial radii and heights at 0.0001 M. The zeta potentials of sand were taken as those of the negatively charged planar surface and the negatively charged asperity. The positively charged asperity was assumed to have zeta potentials same as those of alumina in Fuerstenau and Pradip [50] (see Table 1). Fig 5 shows that the ranges of the asperity radii and asperity heights that can cause the shallow primary energy wells (e.g., < 5 *k*T) are decreased if the asperity surface is positively charged. Therefore, detachment from primary minima will be decreased by the presence of chemical heterogeneity.

**Fig 6:**
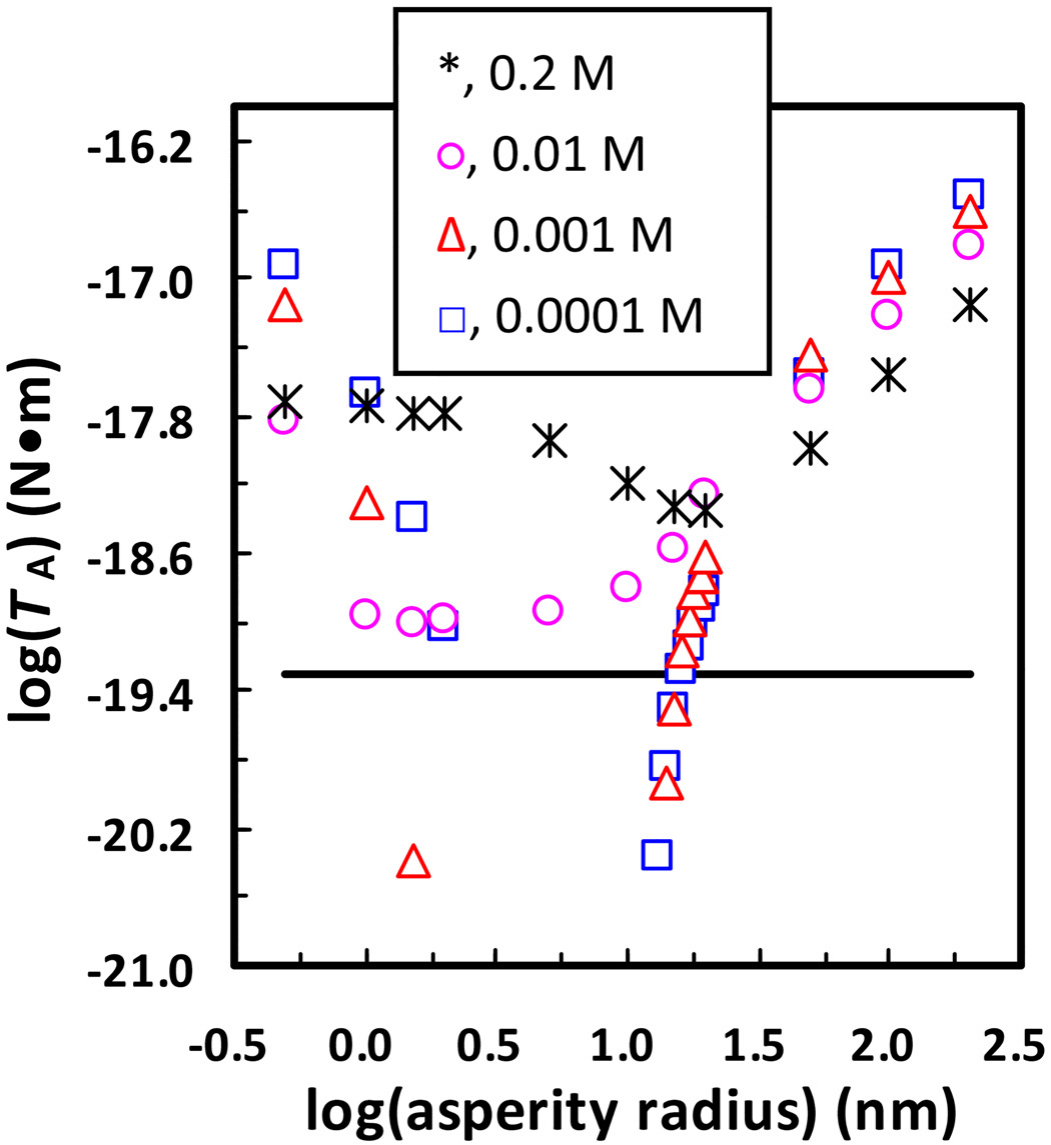
Calculated adhesive torque for the 1156 nm colloid attached atop the hemispherical asperity in Fig 2 as a function of the asperity’s radius at different ionic strengths (□, 0.0001 M; Δ, 0.001 M; ○, 0.01 M; *, 0.2 M). The maximum hydrodynamic torques for approach velocity of 1.2 × 10^−5^ m/s (solid line) was also shown for comparison.

It is worthwhile mentioning that the aforementioned results were obtained by considering the Brownian diffusion as the dominant force for detachment of the 1156 nm colloid. This corresponds to the detachment of the colloid located near the front and rear stagnation point regions of a porous media where the hydrodynamic drag (*T*_H_) effect is minor [51]. The colloid experiences greater hydrodynamic drag if it is attached closer to the midpoint regions of a porous media [52]. To examine whether the 1156 nm colloid can be detached by hydrodynamic drag in our column experiments, the adhesive torques (*T*_A_) that the colloid experiences atop a hemispherical asperity with different radii at different ionic strengths were calculated and are presented in Fig 6. The maximum hydrodynamic torque (i.e., the hydrodynamic drag that the colloid experiences at the midpoint regions) for the approach velocity used in the column experiments is also shown for comparison. Details about the methods used to calculate the adhesive and hydrodynamic torques are given in the Text A in S1 File. Fig 6 shows that even the minimum adhesive torque is greater than the maximum hydrodynamic torque at ≥ 0.01 M. In contrast, there are ranges of asperity radii at which the adhesive torques are smaller than the maximum hydrodynamic torque at ≤ 0.001 M. For example, the adhesive torque that acts on the 1156 nm colloid atop the hemispherical asperity with radius of 15 nm is 3.1 × 10^−20^ N•m at 0.0001 M, which is smaller than the maximum hydrodynamic torque (5.0 × 10^−20^ N•m). Hence, if the asperity is located near the midpoint regions, the colloid initially attached at 0.2 M atop the asperity will be detached at 0.0001 M by hydrodynamic drag although a primary energy well (2.1 *k*T) is still present at 0.0001 M. When the asperity radius is increased to 18 nm, Brownian diffusion, rather than the hydrodynamic torque, will control detachment of the colloid from the primary energy well (4.4 *k*T) even if the asperity is located at the midpoint region due to dominance of the adhesive torque (9.7 × 10^−20^ N•m).

While it has been widely recognized that colloids attached at primary minima can be detached by perturbations in solution chemistry or hydrodynamics [16–19,53–55], the aforementioned theoretical calculations show that colloids can also be spontaneously detached from primary minima by Brownian diffusion under *constant* physiochemical conditions. As will be shown later in the paper, the theoretical prediction is consistent with the column experimental results in this study and additional observations reported in the literature [1,9,12,16,20–22]. Whereas spontaneous detachment is frequently attributed to attachment in secondary minima [13,20,26,56–59], our results indicate that the detached colloids are not necessarily initially attached at secondary minima.

### 4.2. Column breakthrough curves

**Fig 7:**
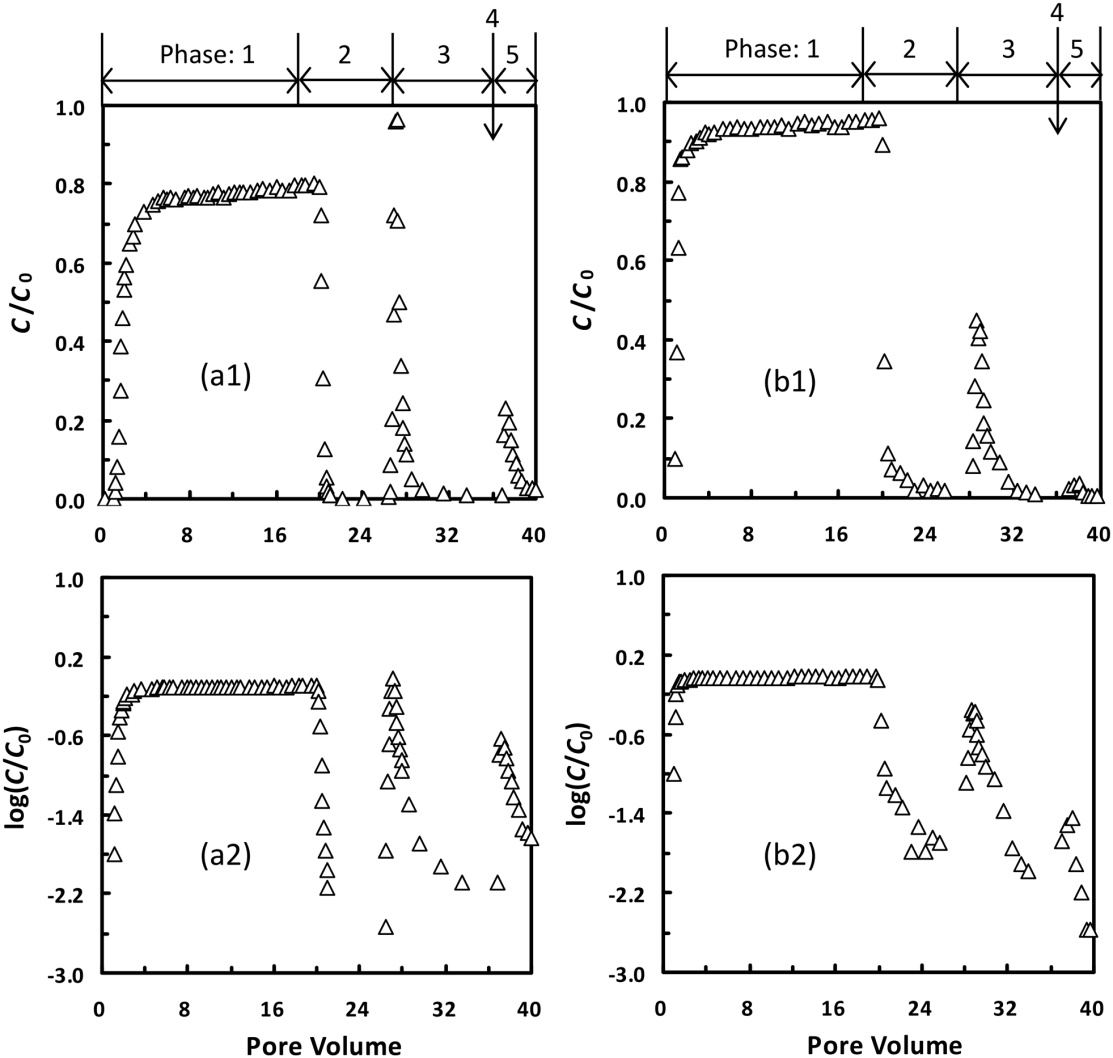
Effluent concentrations for the 1156 nm latex particles from the columns. Phase 1, attachment of colloids at (a) 0.2 M or (b) 0.01 M; Phase 2, elution with colloid-free electrolyte solution; Phase 3, elution with DI water; Phase 4, flow interruption for 3 days; Phase 5, elution with DI water. (2) is re-plotted figure for (1) on a semi-log scale.

Fig 7a presents effluent concentrations for the 1156 nm latex colloids in the columns. In phase 1, the colloids were attached in both primary and secondary minima at 0.2 M according to the aforementioned theoretical calculations. In phase 2, the unattached colloids in pore water were displaced by the introduction of colloid-free NaCl solutions. The tail was absent in the breakthrough curve, indicating that spontaneous detachment from primary or secondary minima was absent. This is because even the smallest primary and secondary minimum depths were significantly greater than the average kinetic energy of a colloid at 0.2 M according to the theoretical calculations in this study and in Shen et al. [26], respectively. In phase 3, all colloids attached at the secondary minima were detached because the introduction of deionized water eliminated secondary minima from the EPs (i.e., type 3 detachment). Colloids attached at primary minima were also detached if the EPs became type III after introducing deionized water (type 2 detachment). The rate of the two types of detachment was not dependent on colloid transport over detachment energy barrier since the energy barrier was absent, but was controlled by transport across the diffusion boundary layer [60]. The type 2 and 3 detachments explain the peak in the breakthrough curve in phase 3 present immediately after introducing deionized water.

Interestingly, Fig 7a shows that a tail was present in phase 3 when the breakthrough curve was plotted on a semi-log scale. The tail indicates the occurrence of energy barrier-controlled detachment, which cannot be explained by the type 2 and 3 detachments. Likewise, the tail in the breakthrough curve cannot be attributed to the spontaneous detachment from secondary minima either because the secondary minima were absent at this ionic strength. Alternatively, the tail can be explained very well by spontaneous detachment from primary minima. Similar tailing phenomenon in the breakthrough curves when deionized water is used to detach colloids has also been observed in the literature [1,9,12,16,20–22,61]. To further confirm that colloids can be spontaneously detached from primary minima, flow of the column system was halted for 3 days in phase 4. The flow interruption lasted for a long period because the spontaneous detachment is a rate-limited process. The 1156 nm colloids were immediately detected from the effluents in phase 5 following re-introduction of deionized water, confirming the presence of spontaneous detachment from primary minima in phase 4.

Fig 7b presents a breakthrough curve from a column experiment following the same sequence of attachment and detachment as those used in Fig 7a except that 0.01 M NaCl was adopted as the background solution instead of 0.2 M NaCl. The spontaneously detached colloids were less in phase 5 when the colloids were initially attached at the lower ionic strength in phase 1. This is expected because a number of asperities that caused colloid attachment at 0.2 M in phase 1 and subsequently spontaneous detachment at 0.0001 M became unavailable for colloid attachment at 0.01 M. For example, the 1156 nm colloid could be attached atop of the hemispherical asperity of 20 nm radius via primary-minimum association at 0.2 M and the attached colloid would be spontaneously detached from primary minima when the ionic strength was decreased to 0.0001 M. The colloid, however, could not be attached atop of the 20 nm hemispherical asperity at 0.01 M due to the presence of a significant energy barrier (19 *k*T). When colloid-free NaCl electrolyte was introduced in phase 1 (i.e., control experiments), detachment was negligible in phase 5 (data not shown), indicating that the influence of colloidal impurities in sand was minor. As shown in the section of Materials and Methods, the sand was extensively treated to remove potential colloidal impurities.

**Fig 8:**
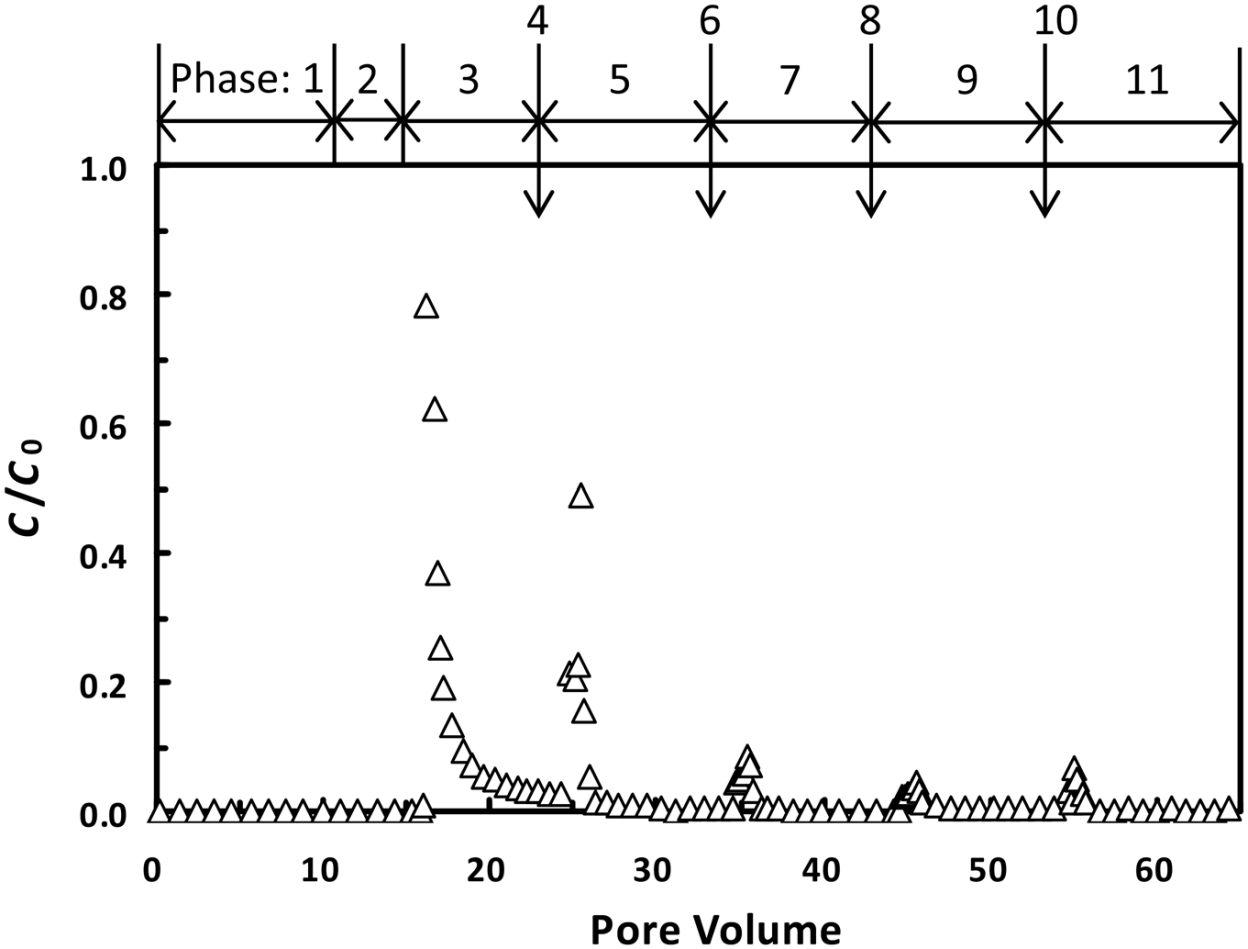
Effluent concentrations for the 133 nm fullerene C_60_ nanoparticles from the columns. Phase 1, attachment of nanoparticles at 0.01 M; Phase 2, elution with colloid-free electrolyte solution; Phase 3, elution with DI water; Phase 4, flow interruption for 1 days; Phase 5, elution with DI water; Phase 6, flow interruption for 2 days; Phase 7, elution with DI water; Phase 8, flow interruption for 3 days; Phase 9, elution with DI water; Phase 10, flow interruption for 4 days; Phase 11, elution with DI water.

Although the results in Fig 7 were obtained for the latex particles whose surface properties are relatively simple, the spontaneous detachment from primary minima could also occur for more complex engineered particles. Fig 8 shows the breakthrough curves for fullerene C_60_ nanoparticles from column experiments conducted following the same procedure as that of the 1156 nm colloids except three more flow interruptions after phase 5. Spontaneous detachment of fullerene nanoparticles from primary minima occurred during each and all flow interruptions, as indicated by the peaks in phases 5, 7, 9, and 11. It should be noted that the theoretical results of our study not only can be used for explaining detachment of exotic colloids (e.g., the latex particles and fullerene nC_60_ nanoparticles), but can also be employed for interpreting detachment of native colloids (e.g., soil clay particles) from sand grains. For example, the clay particles attached on sand grain surfaces via primary-minimum association under drought conditions can be spontaneously detached after rainfall events.

## 5. Discussion

Whereas our theoretical calculations only considered physical heterogeneities on collector surfaces, the presence of physical heterogeneities on colloid surfaces can further decrease depths of primary minima on EPs [62] and accordingly increase spontaneous detachment from primary minima. Our theoretical calculations adopted a low value (i.e., 0.157 nm) for the Born collision parameter to calculate the Born repulsion and a high value (i.e., 1×10^−20^ J) for the Hamaker constant to calculate the van der Waals energy. The spontaneous detachment from primary minima will be more significant if higher values of Born collision parameter and lower values of Hamaker constant are used. To demonstrate this point for a wider range of possible scenarios, Fig C in S1 File compares calculated values of *U*_pri_ for the 1156 nm colloid interacting with the planar surface carrying a hemisphere with different radii at 0.0001 M for Born collision parameter of 0.5 nm and 0.157 nm. The range of asperity radii that can cause shallow primary energy well (e.g., < 5 *k*T) is significantly increased by using 0.5 nm as the Born collision parameter. Similarly, the presence of polymers on latex particle surfaces can cause steric repulsion when the asperities on collector surfaces interact with the polymer coated particle surfaces. By using the method of Kim and Matsen [63] to estimate the steric repulsion energy (see Text B in S1 File), Fig D in S1 File shows that the steric repulsion can significantly decrease the primary minimum depths and accordingly increase spontaneous detachment from primary minima. In contrast, the presence of attractive short-range forces (e.g., hydrophobic and π-π interactions) [64] will decrease the magnitude of spontaneous detachment from primary minima.

Early studies [60,65,66] viewed the primary minimum as the only location for colloid attachment and, in order to explain the observed detachments, concluded that colloids could be detached from primary minima by Brownian diffusion. To obtain a shallow-enough primary energy well and to make colloid detachment Brownian diffusion possible, these studies had to significantly increase the minimum colloid-collector separation distance in the mean-field DLVO approach. Nevertheless, they did not consider the fact that colloids could not be initially attached at large values of minimum colloid-collector separation distance. Therefore, the experimentally observed spontaneous detachments were frequently attributed to the attachment in secondary minima under unfavorable conditions in later studies [13,20,26,56–59]. Our column experiments, however, unambiguously showed that colloids can be spontaneously detached from primary minima by Brownian diffusion under unfavorable conditions.

The previous theoretical calculations show that the variations of height and curvature of discrete physical heterogeneities result in a distribution of primary minimum depths. Fig E in S1 File presented calculated primary minimum depth *U*_pri_ for the 1156 nm colloid interacting with the planar surface where nanoscale pillars are randomly distributed. The total interaction energy *U* was calculated by *U* = (1−*f*)*U*(*H*+*H*_P_) + *fU*(*H*), where *H*_p_ is height of the nanoscale pillars and *f* is density of the pillars [67, 68]. The results show that the distribution of primary minimum depths can also be caused by varying density of discrete physical heterogeneities. Note that Pazmino et al. [54] also showed a distribution of primary minimum depths by considering power law size-distributed discrete chemical heterogeneities on collector surfaces. These theoretical results provide plausible explanations for a distribution of colloid detachment rate coefficients observed in the columns experiments conducted over long-time periods [69,70]. However, it should be noted that the previous study employed the balance of hydrodynamic and adhesive torques as the sole criteria for determining detachment of colloids from primary minima, similar to previous studies [3,5,15,29,31,32,52,71,72]. Our results indicate that this approach could underestimate the detachment of colloids from primary minima because the spontaneous detachment by Brownian diffusion (i.e., the slow detachment stage) was not taken into consideration.

The spontaneous disaggregation from primary minima is more significant than spontaneous detachment from collector surfaces. This is because the DLVO interaction energy between colloid 1 with radius of *a*_p1_ and colloid 2 with radius of *a*_p2_ is *a*_p2_/(*a*_p1_+ *a*_p2_) times the magnitude greater than that between the colloid 1 and a planar collector surface [6,38–40]. This means that the DLVO interaction energy (and accordingly the primary minimum depth) between colloid 1 and the planar surface is the upper bound of the DLVO energies between the colloid 1 and 2. Particularly, when *a*_p1_ is equal to *a*_p2_, the DLVO interaction energy between the two colloids is half of the interaction energy between one of the colloids and a planar surface. Therefore, the presence of discrete physical heterogeneity can cause more shallow primary energy wells between two colloids than between a colloid and a collector. This indicates that colloids aggregated at primary minima could also be spontaneously disaggregated by Brownian diffusion, similar to the spontaneous redispersion of aggregated colloids from secondary minima [73–79].

**Fig 9:**
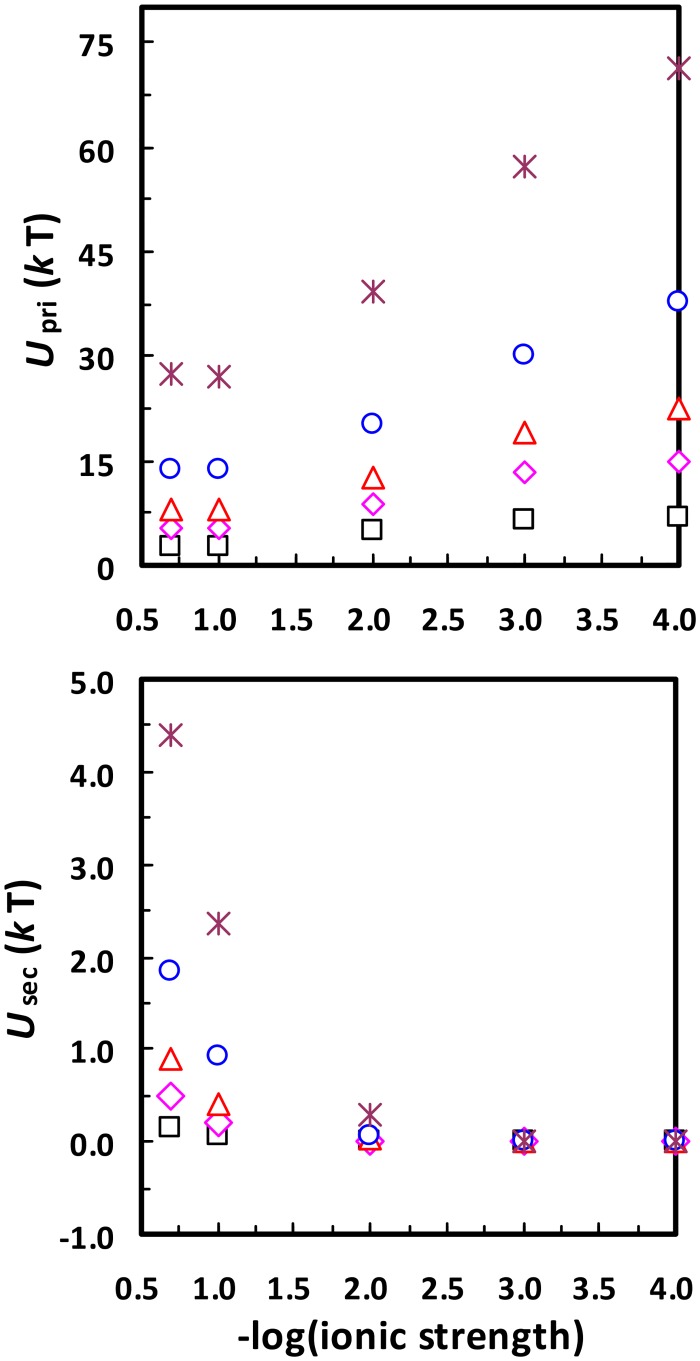
Calculated primary minimum depths (*U*_pri_) and secondary minimum depths (*U*_sec_) between a planar surface and nanoparticles of different radii (□, 10 nm; ◊, 20 nm; Δ, 30 nm; ○, 50 nm; *, 100 nm) at different ionic strengths. The zeta potentials of the nanoparticles and the planar surface were assumed to be the same as those of 1156 nm colloid and sand in Table 1, respectively.

**Fig 10:**
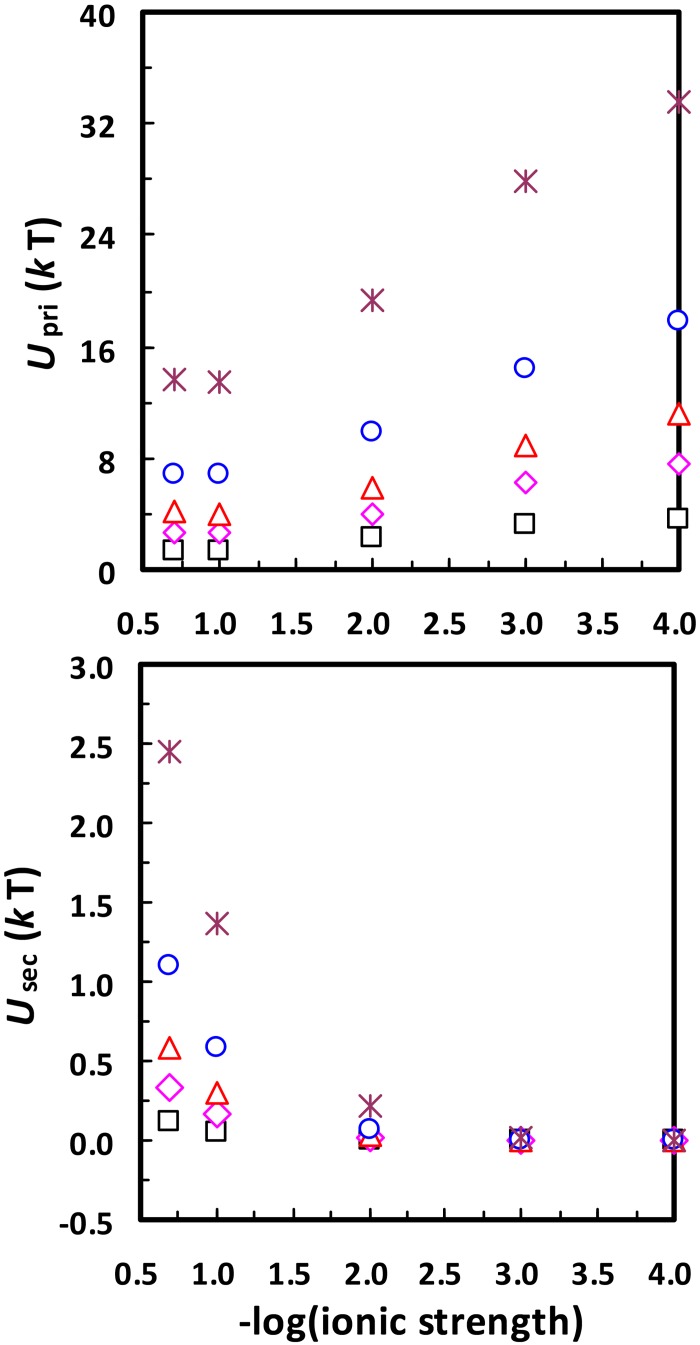
Calculated primary minimum depths (*U*_pri_) and secondary minimum depths (*U*_sec_) between two identical nanoparticles of different radii (□, 10 nm; ◊, 20 nm; Δ, 30 nm; ○, 50 nm; *, 100 nm) at different ionic strengths. The zeta potentials of the nanoparticles were assumed to be the same as those of 1156 nm colloid in Table 1.

The DLVO interaction energy (and accordingly the primary minimum depth) between a colloid and a planar collector surface is proportional to the size of the colloid [6,38–40]. This means that the presence of surface physical heterogeneity can cause more spontaneous disaggregation/detachment from primary minima for aggregated/attached nanoparticles than microparticles. In fact, spontaneous detachment/disaggregation for nanoparticles with sizes < 30 nm (e.g., quantum dots and viruses) [22] is more likely due to release from primary minima than from secondary minima. Specifically, Fig 9 presents calculated secondary minimum depths between a planar surface and a nanoparticle of different radii at different ionic strengths. The secondary minimum depths are much smaller than the average kinetic energy of a colloid for nanoparticles with sizes < 30 nm at all ionic strengths considered. The secondary minimum depth is further decreased if the interaction is between two nanoparticles (see Fig 10). Therefore, the secondary minimum should have minor influence on attachment/aggregation of < 30 nm nanoparticles and the spontaneous detachments observed for these small nanoparticles are more likely due to the release from primary minima.

## 6. Conclusions

A distribution of primary energy minimum depths has been obtained in Pazmino et al. [54] by considering power law size-distributed discrete chemical heterogeneities on collector surfaces. Our study, through evaluating the DLVO interaction energies using grid-surface integration technique [31,32], showed that the variation of height, curvature, and density of discrete physical heterogeneities can also result in a distribution of primary minimum depths. Furthermore, we highlighted the EP with a shallow primary minimum comparable to the average kinetic energy of a colloid and a monotonic decrease of interaction energy with separation distance beyond the primary energy well. The EP indicates that colloids attached at the primary energy well can be spontaneously detached to bulk solution by Brownian diffusion. The column transport experiments involving flow interruptions unambiguously verified the presence of spontaneous detachment from primary minima without perturbations in solution chemistry or hydrodynamics under unfavorable conditions for both model colloids (i.e., polystyrene latex microspheres) and engineered nanoparticles (i.e., fullerene C_60_ aggregates). Whereas the spontaneous detachment is frequently attributed to attachment in secondary minima, our theoretical and experimental results indicate that the detached colloids are not necessarily initially attached at the secondary minima. Our study illustrates the limitation of using a balance of hydrodynamic and adhesive torques as the sole criteria for determining detachment of colloids from primary minima.

Spontaneous detachment from primary minima is likely more significant for nanoparticles than microparticles because the DLVO interaction energies and accordingly the primary minimum depths are lower for smaller colloids. Similarly, the aggregated colloids at primary minima may be more readily disaggregated by Brownian diffusion compared to the spontaneous detachment of colloids from collector surfaces. These results imply that the slow process of spontaneous detachment/disaggregation of colloids from primary minima must be considered in theoretical models for accurately predicting the fate and transport of colloids, especially nanoparticles, in subsurface environments.

## Supporting Information

S1 File Torque analysis (Text A). Calculation of steric repulsion energy (Text B). Expressions for calculating *E*^VDW^, *E*^DL^, and *E*^BR^ differential interaction energies (Table A in S1 File). Zeta potential for the 1156 nm colloid as a function of pH at ionic strengths of 0.01 M and 0.2 M (Fig A in S1 File). Calculated primary minimum depth *U*_pri_ for the 1156 nm colloid interacting with the planar surface carrying a hemispheroid as a function of equatorial radius for various hemispheroid heights (Δ, 2 nm; □, 5 nm; ◊, 10 nm; ○, 20 nm; *, 100 nm) at different ionic strengths (a, 0.0001 M; b, 0.001 M; c, 0.01 M; d, 0.2 M) (Fig B in S1 File). Calculated primary minimum depths *U*_pri_ for the 1156 nm colloid interacting with the planar surface carrying a hemisphere with different radii at ionic strength of 0.0001 M for Born collision parameter of (1) 0.157 nm and (2) 0.5 nm (Fig C in S1 File). Calculated primary minimum depths *U*_pri_ for the 1156 nm colloid (a) with and (b) without a polymer layer interacting with the planar surface carrying a hemisphere with different radii at ionic strength of 0.0001 M (Fig D in S1 File). Calculated primary minimum depths *U*_pri_ for the 1156 nm colloid interacting with the planar surface carrying nanoscale pillars with different heights (*H*_p_) and densities (*f*) (Fig E in S1 File).
(DOC)
