# Supplementary material for: Spontaneous Detachment of Colloids from Primary Energy Minima by Brownian Diffusion
Source: PLoS One. 2016 Jan 19;11(1):e0147368. doi: 10.1371/journal.pone.0147368 (PMC4718715; doi:10.1371/journal.pone.0147368)
Supplement: S1 File — Expressions for calculating EVDW, EDL, and EBR differential interaction energies (Table A in S1 File). Zeta potential for the 1156 nm colloid as a function of pH at ionic strengths of 0.01 M and 0.2 M (Fig A in S1 File). Calculated primary minimum depth Upri for the 1156 nm colloid interacting with the planar surface carrying a hemispheroid as a function of equatorial radius for various hemispheroid heights (Δ, 2 nm; □, 5 nm; ◊, 10 nm; ○, 20 nm; *, 100 nm) at different ionic strengths (a, 0.0001 M; b, 0.001 M; c, 0.01 M; d, 0.2 M) (Fig B in S1 File). Calculated primary minimum depths Upri for the 1156 nm colloid interacting with the planar surface carrying a hemisphere with different radii at ionic strength of 0.0001 M for Born collision parameter of (1) 0.157 nm and (2) 0.5 nm (Fig C in S1 File). Calculated primary minimum depths Upri for the 1156 nm colloid (a) with and (b) without a polymer layer interacting with the planar surface carrying a hemisphere with different radii at ionic strength of 0.0001 M (Fig D in S1 File). Calculated primary minimum depths Upri for the 1156 nm colloid interacting with the planar surface carrying nanoscale pillars with different heights (Hp) and densities (f) (Fig E in S1 File). (DOC) [file pone.0147368.s001.doc]

Supporting Information

Spontaneous Detachment of Colloids from Primary Energy Minima by Brownian Diffusion

Zhan Wang^1,2^, Yan Jin^3^, Chongyang Shen^1,*^, Tiantian Li^1^, Yuanfang Huang^1^, Baoguo Li^1,*^

^1^Department of Soil and Water Sciences, China Agricultural University, Beijing 100193, China.

^2^College of Land and Environment, Shenyang Agricultural Universtiy, Shenyang, Liaoning 110866, China.

^3^Department of Plant and Soil Sciences, University of Delaware, Newark, Delaware 19716, United States.

^*^Corresponding author, Email: chongyang.shen@gmail.com (CS); libg@cau.edu.cn (BL).

**Text A. Torque analysis**

The adhesive torque (*T*_A_) is represented by the adhesive force (*F*_A_) acting on a lever arm *l*_c_:

$T_{\text{A}}=F_{\text{A}}l_{\text{c}}$ (S1)

The adhesive forces were taken as the maximum attraction of DLVO interaction force curves [1]. Specifically, the value of adhesive force was estimated as *U*_pri_/*H*_pri_ for colloids attached at primary minimum, where *H*_pri_ is separation distance of the primary minimum. The lever arm is provided by the radius of particle-surface contact area (*a*_0_), resulting from deformation of the particle. The equation that was used to calculate the radius of particle-surface contact area for a colloid atop of a hemispherical asperity is written as

$a_{0}=\left(\frac{3F_{\text{A}}a_{\text{p}}a_{g}}{4E\left(a_{p}+a_{g}\right)}\right)^{1/3}$ (S2)

where *a*_p_ is radius of a colloid; *a*_g_ is radius of asperity; *E* is elastic interaction constant. A value of 4.0×10^9^ N m^-2^ was taken as the elastic interaction constant for the quartz collectors and polystyrene particle suspensions [2,3]. The hydrodynamic torque (*T*_H_) experienced by a colloid in the vicinity of the collector surface due to hydrodynamic shear is expressed as [3,4]

$T_{\text{H}}=1.4a_{\text{p}}F_{\text{H}}$ (S3)

where *F*_H_ is the drag force experienced by an attached particle in a laminar flow field, which is written as [4]

$F_{\text{H}}=10.205\pi\mu\left(\partial V/\partial r\right)a_{\text{p}}^{2}$ (S4)

where µ is fluid viscosity and ∂*V*/∂*r* is hydrodynamic shear. The expression of ∂*V*/∂*r* for the Happel’s sphere-in-cell model is [5]

$\partial V/\partial r=\frac{1}{2}Ua_{\text{c}}^{2}\text{sin}\theta\left[3K_{1}\left(a_{\text{c}}/r^{4}\right)-K_{2}\left(1/(a_{\text{c}}r^{2})\right)+8K_{4}r/a_{\text{c}}^{4}\right]$ (S5)

where *U* is Darcy velocity; *a*_c_ is radius of a collector; *θ* is tangential coordinate; *r* is a radial coordinate originating from the center of the spherical collector; *K*_1_, *K*_2_, and *K*_4_ are functions that depend on the porosity of the porous media and their definition can be found in Elimelech [6].

**Text B. Calculation of steric repulsion energy**

When a polymer coated particle approach the rough collector surface in Figure 2, the asperity can penetrate into the polymer layer and cause steric repulsion. The steric repulsion *U*^ST^ was estimated by the following equation [7]

$U^{\text{ST}}=\frac{a_{\text{p}}a_{\text{g}}}{a_{\text{p}}+a_{\text{g}}}\frac{9\pi^{3}\sigma L_{0}^{3}}{20L_{\text{a}}^{2}N}\left(\text{-45-30ln}\left(H/L_{0}\right)+54\left(H/L_{0}\right)-10\left(H/L_{0}\right)^{3}+\left(H/L_{0}\right)^{6}\right)/54$ (S6)

where *σ* is grafting density of the polymer layer, *L*_0_ is thickness of the polymer layer, *L*_a_ is statistic segment length, *N* is the number of segments per chain, *H* is separation distance between the asperity and the particle.

Table A. Expressions for calculating *E*^VDW^, *E*^DL^, and *E*^BR^ differential interaction energies.

| type of interaction | expressions |
| --- | --- |
| VDW | $E^{\text{VDW}}\left(h\right)=-\frac{A_{\text{H}}}{12\pi h^{2}}$ |
| DL | $E^{\text{DL}}\left(h\right)=32\varepsilon\varepsilon_{0}\kappa\tanh\left(\frac{ve\psi_{1}}{4kT}\right)\tanh\left(\frac{ve\psi_{2}}{4kT}\right)\left(\frac{kT}{ve}\right)^{2}\exp\left(-\kappa h\right)$ |
| BR | $E^{\text{BR}}\left(h\right)=-\frac{A_{\text{H}}H_{0}^{6}}{48\pi h^{8}}$ |

*A*_H_ is the Hamaker constant, *h* is the separation distance between a surface element on a nC_60_ NP surface and the collector surface, *ɛ*_0_ is the dielectric permittivity in vacuum, *ɛ* is the relative dielectric permittivity of solvent, κ is the inverse Debye screening length, *v* is the charge number, *e* is the electronic charge *ψ*_1_ and *ψ*_2_ are the surface potentials of nC_60_ colloid and collector, respectively, and *H*_0_ is minimum separation distance between the nC_60_ NP and collector, taken as 0.158 nm [8].

**References**

1. Sharma P, Flury M, Zhou J. Detachment of colloids from a solid surface by a moving air-water interface. J Colloid Interface Sci. 2008; 326: 143-150.
2. Bergendahl JA, Grasso D. Prediction of colloid detachment in a model porous media: Hydrodynamics. Chem Eng Sci. 2000; 55: 1523-1532.
3. Torkzaban S, Bradford SA, Walker SL. Resolving the coupled effects of hydrodynamics and DLVO forces on colloid attachment in porous media. Langmuir 2007; 23: 9652-9660.
4. O’Neill ME. A sphere in contact with a plane wall in a slow linear shear flow. Chem Eng Sci. 1968; 23: 1293-1298.
5. Shen C, Huang Y, Li B, Jin Y. Predicting attachment efficiency of colloid deposition under unfavorable attachment conditions. Water Resour Res. 2010; 46: W11526. doi:10.1029/2010WR009218.
6. Elimelech M. Particle deposition on ideal collectors from dilute flowing suspensions: Mathematic formulation, numerical solution, and simulations. Sep Technol. 1994; 4: 186-212.
7. Kim JU, Matsen MW. Repulsion exerted on a spherical particle by a polymer brush. Macromolecules 2008; 41: 246-252.
8. Wang H, Zhang Newby B. Applicability of the extended Derjaguin-Landau-Verwey-Overbeek theory on the adsorption of bovine serum albumin on solid surfaces. Biointerphases 2014; 9: 041006.

∆, 0.01 M

*, 0.2 M

Fig A. Zeta potential for the 1156 nm colloid as a function of pH at ionic strengths of 0.01 M and 0.2 M.

(a)

(b)

(c)

(d)

Fig B. Calculated primary minimum depth *U*_pri_ for the 1156 nm colloid interacting with the planar surface carrying a hemispheroid as a function of equatorial radius for various hemispheroid heights (Δ, 2 nm; □, 5 nm; ◊, 10 nm; ○, 20 nm; *, 100 nm) at different ionic strengths (a, 0.0001 M; b, 0.001 M; c, 0.01 M; d, 0.2 M). Inserts are re-plotted profiles in a different scale of the *y* axis.

(a1)

(b1)

(a2)

(b2)

Fig C. Calculated primary minimum depths *U*_pri_ for the 1156 nm colloid interacting with the planar surface carrying a hemisphere with different radii at ionic strength of 0.0001 M for Born collision parameter of (1) 0.157 nm and (2) 0.5 nm. (b) are replotted figures in a different scale of the *y* axis for (a) to highlight the shallow primary energy wells.

(a)

(b)

Fig D. Calculated primary minimum depths *U*_pri_ for the 1156 nm colloid (a) with and (b) without a polymer layer interacting with the planar surface carrying a hemisphere with different radii at ionic strength of 0.0001 M. σ = 1 nm^2^, *L*_0_ = *L*_a_*N*^1/2^ = 1 nm.

∆, *H*_p_=2 nm

○, *H*_p_=5 nm

*, *H*_p_=50 nm

Fig E. Calculated primary minimum depths *U*_pri_ for the 1156 nm colloid interacting with the planar surface carrying nanoscale pillars with different heights (*H*_p_) and densities (*f*).
